# Evolution of antero‐posterior patterning of the limb: Insights from the chick

**DOI:** 10.1002/dvg.23047

**Published:** 2017-07-22

**Authors:** Matthew Towers

**Affiliations:** ^1^ Department of Biomedical Science The Bateson Centre, University of Sheffield Western Bank Sheffield S10 2TN United Kingdom

**Keywords:** avian, chick, digits, dinosaur, limb, positional information, self organization, Shh, theropod, tetrapod

## Abstract

The developing limbs of chicken embryos have served as pioneering models for understanding pattern formation for over a century. The ease with which chick wing and leg buds can be experimentally manipulated, while the embryo is still in the egg, has resulted in the discovery of important developmental organisers, and subsequently, the signals that they produce. Sonic hedgehog (Shh) is produced by mesenchyme cells of the polarizing region at the posterior margin of the limb bud and specifies positional values across the antero‐posterior axis (the axis running from the thumb to the little finger). Detailed experimental embryology has revealed the fundamental parameters required to specify antero‐posterior positional values in response to Shh signaling in chick wing and leg buds. In this review, the evolution of the avian wing and leg will be discussed in the broad context of tetrapod paleontology, and more specifically, ancestral theropod dinosaur paleontology. How the parameters that dictate antero‐posterior patterning could have been modulated to produce the avian wing and leg digit patterns will be considered. Finally, broader speculations will be made regarding what the antero‐posterior patterning of chick limbs can tell us about the evolution of other digit patterns, including those that were found in the limbs of the earliest tetrapods.

## 1 INTRODUCTION

Understanding how the embryonic limb is patterned has intrigued generations of researchers. One reason for this, apart from the tractability of the limb as an experimental system, is that the limb fascinates us in having such diverse forms—a consequence of its repeated modification and selection during the course of evolution to suit the functional needs of a given species (Saxena, Towers, & Cooper, 2017). It is the digits of the limb that have undergone the most extensive modification during evolution, both in terms of the number that form, and in their anatomies, such as the number of phalanges that they have. Indeed, the model species that are generally used to dissect the mechanisms of limb pattern formation—commonly the chick and mouse—have very different digit patterns. In addition, the techniques that researchers use to address the questions of limb pattern formation are often diverse—mostly traditional experimental embryology in the chick, and mostly genetics in the mouse. This has made it difficult to understand how anatomically distinct digit patterns have evolved. In this review, it will be discussed if theoretical models, which have resulted from decades of embryological research on chick limbs, can enlighten us about how the avian wing and leg digit patterns evolved. The chick leg digit pattern, in having remained relatively unchanged throughout tetrapod evolution, will be highlighted as it presents a unique opportunity to understand how the ancestral amniote limb was patterned. Based on this, speculations will be made about how such a patterning mechanism could have arisen and then how it could have been subsequently adapted in different tetrapod lineages.

### 1.1 General trends in the evolution of digit pattern

**Figure 1:**
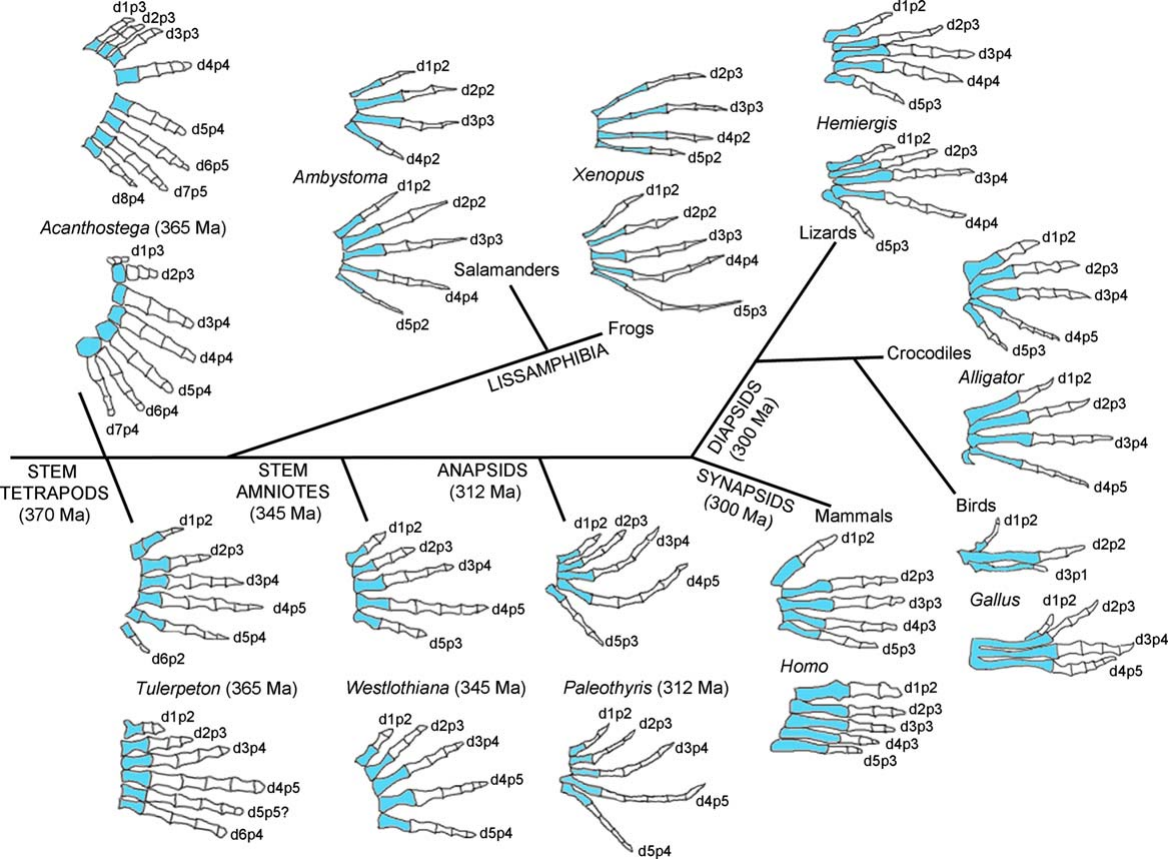
General trends in the evolution of tetrapod digit patterns. Fore‐limb digit (d) patterns (upper) and hind‐limb digit patterns (lower) of the limbs of species from a selection of tetrapod groups. In all cases, white elements are the phalanges (p) and their number is shown; blue elements are metacarpals/carpals or metatarsals. The numbering of digits reflects known patterns of digit loss, for example, digit 1 in *Xenopus* fore‐limbs. Digit patterns of extinct species drawn after (Coates and Clack, 1990) (*Acanthosteg*a); (Lebedev and Coates, 1995) (*Tulerpeton*); (Smithson et al., 1994) (*Westlothiana*); (Carroll, 1969) (*Paleothyris*). Ma is millions of years ago

For many developmental biologists who have not studied the fossil record, it is a surprise to learn that the limbs of stem tetrapods that existed during the late Devonian period were polydactylous (having more than five digits). This can be appreciated in the paddle‐like limbs of *Acanthostega*: its fore‐limbs had eight digits and its hind‐limbs had seven digits, with the number of phalanges per digit in both fore‐limbs and hind‐limbs ranging from three to five (Figure 1, Clack, 2002; Coates & Clack, 1990). It is worth noting that in *Acanthostega* limbs, digits with the same number of phalanges were generally found together; when the phalangeal count changed between two adjacent digits, this was always by one, and the number of phalanges increased in the digits running from anterior to posterior—except in the most‐posterior digit of the fore‐limb (Figure 1). These characteristics can often be recognized in the fore‐limbs and hind‐limbs of many contemporary tetrapods and the potential relevance of this will be discussed in the final section. Like *Acanthostega*, *Tulerpeton* was a stem tetrapod, but it had six digits in both its fore‐limbs and hind‐limbs, and is significant because it is one of the earliest examples in which the basal amniote phalangeal count in digits 1, 2, 3, and 4 can be observed (Lebedev, 1990; Lebedev & Coates, 1995), having 2, 3, 4, 5 phalanges, respectively (Romer, 1956). In addition, the limbs of *Tulerpeton* are among the earliest known to have biphalangeal anterior “thumb” digits—a defining character of the limbs of many later tetrapods. Although the fossil record is sparse for the period running from the late Devonian into the Carboniferous, during a roughly 15 million year period known as Romer's Gap (360–345 Ma), recently unearthed fossils have started to reveal how the polydactylous limbs of early tetrapods evolved into the pentadactyl limbs of the first amniotes (Clack, 2002). The stabilization of pentadactyly can be observed in the limbs of the important fossil, *Westlothiana*, that has been classified as a stem amniote (Smithson, Carroll, Panchen, & Andrews, 1994). Rather confusingly, stem amniote is a loose term that includes animals that are not necessarily viewed as amniotes, such as anthracosaurs, but excludes the lissamphibia (Figure 1, reviewed in Clack, 2012). However, in terms of limb evolution, *Westlothiana* is pivotal, since it exhibited the basal amniote phalangeal count in its fore‐limbs and hind‐limbs (2‐3‐4‐5‐3 in the fore‐limb, and 2–3‐4–5‐4 in the hind‐limb, Smithson et al., 1994) —patterns that were common to the limbs of the first definitive anapsid amniotes such as *Paleothyris* (Figure 1; Carroll, 1969).

At the end of the Carboniferous period, two major groups of amniotes diverged: the synapsids, which gave rise to mammals; and the diapsids, which gave rise to lizards, snakes, crocodiles and dinosaurs/birds, among others. The limbs of many mammalian species have undergone digit loss (ungulates such as pigs, horses, cows and rodents such as jerboas, to name but a few), the basis of which we are beginning to understand (reviewed in Saxena et al., 2017). An early event in the evolution of synapsid limbs was a reduction in the number of phalanges in digits 3, 4 and 5 in both fore‐limbs and hind‐limbs, to make the general mammalian phalangeal pattern (2–3‐3–3‐3; Hopson, 1995), which can be observed in the digits of our own limbs (Figure 1), as well as in the digits of the limbs of many other contemporary species. Again, there are notable examples of further digit loss in diapsid lizards, such as in Australian skinks (Shapiro, 2002), and even limb loss altogether in snakes, for example. However, it is worth pointing out that the limbs of some contemporary diapsids, including alligators and lizards, still display a basal amniote phalangeal formula in digits 1, 2, 3 and 4. This is of interest because it suggests that mechanisms that pattern the digits of these limbs have been conserved for some considerable time. As will be discussed in section 1.4, the bird leg also has the basal amniote phalangeal formula in digits 1, 2, 3, and 4, but has lost digit 5. However, the bird wing, in being reduced to three digits, in which two of the remaining digits have lost phalanges, has been transformed into a very specialized pattern over the course of its evolution. The next section will discuss what the fossil record can tell us about bird wing evolution.

### 1.2 Evolution of theropod dinosaur/bird limbs

**Figure 2:**
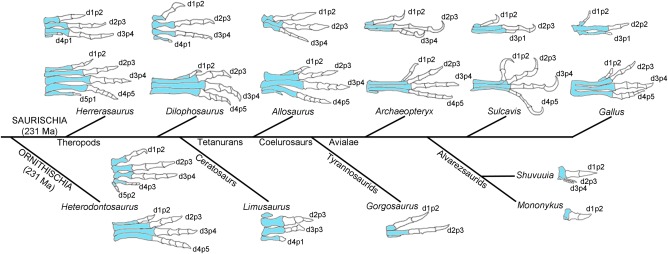
Evolution of theropod dinosaur/bird limbs. Fore‐limb digit (d) patterns (upper) and hind‐limb digit patterns (lower) of a selection of dinosaurs and birds. In all cases, white elements are the phalanges (p) and their number is shown; blue elements are metacarpals or metatarsals. Digit patterns of extinct species drawn after (Sereno and Novas, 1992) (*Herrerasaurus*); (Lebedev and Coates, 1995) (*Heterodontosaurus*); (Welles, 1984) (*Dilophosaurus*); (Madsen, 1976) (*Allosaurus*); (Xu et al., 2009) (*Limusaurus*); (Lambe, 1917) (*Gorgosaurus*); (Ostrom, 1976) (*Archaeopteryx*); (Xu et al., 2011) (*Shuvuuia*); (Xu et al., 2011) (*Mononykus*); (O'connor et al., 2013) (*Sulcavis*). Ma is millions of years ago

The idea that birds evolved from bipedal theropod dinosaurs is now widely accepted (Padian & Chiappe, 1998; Prum, 2002), but was for a long time one of several competing theories (see Feduccia, 2002). Dinosaurs evolved from primitive diapsid reptiles called archosaurs—a group that also gave rise to pterosaurs and today's crocodiles (Benton, 2004). During the radiation of the earliest dinosaurs in the late Triassic, two major groups diverged: the ornithischians, which included *Heterodontosaurus*; and the saurischians, which included one of the earliest putative theropods, *Herrerasaurus* (Figure 2, Benton, 2004—note the phylogenetic position of *Herrerasaurus* has been recently debated, Baron et al., 2017). In the fossils of both animals, the loss or reduction of posterior digits is evident in both pairs of limbs, indicating that these patterning changes had commenced in their common archosaur ancestor (Figure 2, Sereno, 2012; Sereno & Novas, 1992). In the fore‐limbs of *Herrerasaurus*, digit 5 was considerably reduced, leaving a single metacarpal at its base, and digit 4 had a single phalanx (Figure 2); while in its hind‐limbs, digit 5 was absent (Figure 2, Sereno & Novas, 1992). A similar pattern can be seen in the fore‐limbs of the later theropod, *Dilophosaurus* (Figure 2, Welles, 1984), which is thought to have been a close ancestor of two major groups of theropods—the tetanurans, which gave rise to birds, and the ceratosaurs, which included some unusual theropods (Figure 2, Benton, 2004). One being *Limusaurus*, which has received considerable attention for having undergone an unusual pattern of digit loss for a theropod, in which it appears to have lost both anterior and posterior structures in its fore‐limbs: digit 1 was reduced to a single metacarpal and digit 4 had only one phalanx (Figure 2—see section 1.7, Xu et al., 2009). However, *Limusaurus* appears to be very much an outlier in the evolutionary history of birds, rather than a transitional species. Indeed, *Ceratosaurus*, a basal ceratosaur, possessed hands very similar to the basal tetrapod *Dilophosaurus*, suggesting that the hands of *Limusaurus* were derived (Guinard, 2016). If we concentrate on the tetanuran lineage that gave rise to birds, *Allosaurus* was a late Jurassic theropod that had completely lost digit 4 in its fore‐limbs, while digit 5 was reduced to a single metatarsal in its hind‐limbs (Figure 2, Madsen, 1976). Throughout the evolution of the next major group of tetanuran theropods—the coelurosaurs—representative species became more bird‐like, both in having feathers, and in becoming smaller. Of interest are members of some groups of coelurosaurs that showed further reductions in the numbers of elements in their fore‐limbs. Some tyrannosaurids, including *Tyrannosaurus* and *Gorgosaurus*, possessed only two distinct digits, 1 and 2, and their third digits were reduced to a single metacarpal (Figure 2 shows *Gorgosaurus* fore‐limbs, Lambe, 1917). Even more dramatic were the hands of some members of the bird‐like alvarezsaurs*: Shuvuuia* had a large anterior digit 1, and two extremely tiny digits, 2 and 3, which retained the ancestral phalangeal pattern; *Mononyku*s also had a relatively large digit 1, and its two adjacent digits, 2 and 3, were each composed of a single rudimentary metacarpal (Figure 2, Xu et al., 2011). Some of these extreme cases of skeletal element reduction will be considered in section 1.7. However, it is in the avialae in which the true transitional forms that straddle dinosaurs and birds existed, such as the famous *Archaeoptery*x, whose fore‐limbs were used to support the direct ancestry of dinosaurs and birds (Figure 2, (Ostrom, 1976; Zhou, 2004). One reason for this was the similar fore‐limb and hind‐limb anatomies that *Archaeopteryx* shared with theropods such as *Deinonychus* and *Allosaurus* (Figure 2). Later enantiornithine birds, such as *Sulcavis*, also had three wing digits, with the third being reduced to a single phalanx and a metacarpal; the leg had four digits (Figure 2, O'connor et al., 2013). Similar digit patterns are seen in the limbs of most modern birds. The chicken (*Gallus*) is shown as an example of a modern bird, and is of interest because the wing has only two phalanges in its middle digit, although it has been proposed that it could have three phalanges as an embryo, two of which fuse (Figure 2, Seki et al., 2012). Therefore, during the transition from theropod dinosaurs to modern birds, the number of posterior structures was reduced in the fore‐limb—adaptations that are likely to have facilitated flight. By contrast, the four remaining digits of the hind‐limb have retained the basal amniote phalangeal pattern, first seen in stem amniotes (Figure 1).

### 1.3 Chick wing digit patterning

In order to speculate upon the evolutionary changes that have resulted in the formation of a particular pattern, it is imperative to understand the mechanisms that specify this pattern. This has been advanced by decades of experimental embryology on chick limbs, often involving “cut and paste” grafting procedures, as well as, the creation of cellular fate maps. Such experimentation led to the discovery of important signaling centers that influence pattern formation along the different developmental axes (antero‐posterior—thumb to little finger; proximo‐distal—shoulder to digits and dorso‐ventral—back of the hand to palm, reviewed in Tickle, 2017)

**Figure 3:**
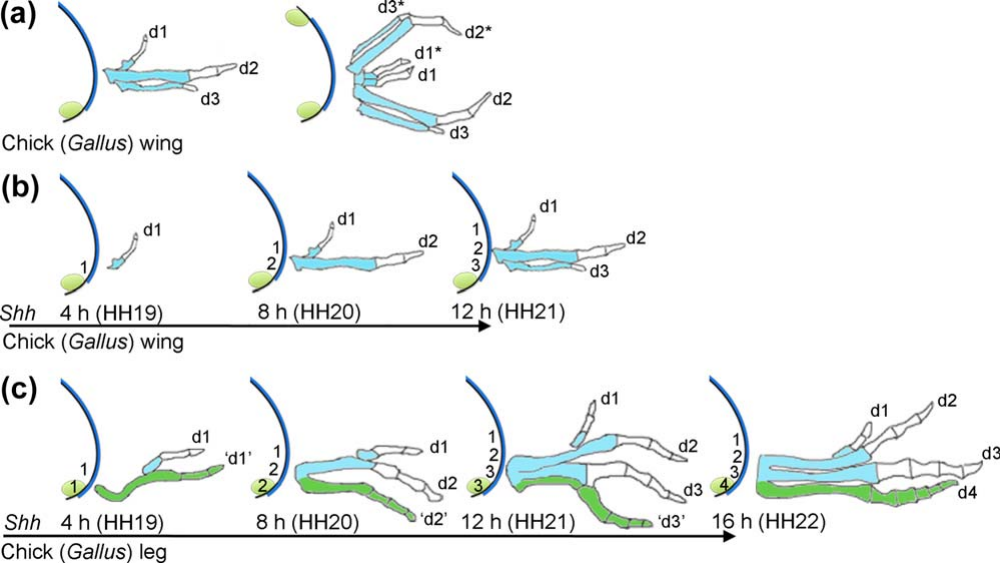
Chick limb digit patterning. **(a**) Limb bud showing polarizing region (green) and apical ectodermal ridge (blue). Grafts of a chick wing polarizing region made to the anterior margin of a second bud fully duplicate the normal pattern of three digits in mirror symmetry (duplicated digits show by asterisk). (**b**) Chick wing antero‐posterior specification—paracrine Shh signaling forms a concentration gradient from the polarizing region and specifies antero‐posterior positional values in cells adjacent to the polarizing region over 12 h. Cells are first specified with anterior positional values (appropriate to specify a digit 1) and are then promoted through more‐posterior positional values (appropriate to specify a digit 2 and then a digit 3) —digit condensations form at later stages by self‐organization. Digit patterns shown are obtained if cyclopamine is added at the Hamburger Hamilton stage of development indicated (shown also in hours of *Shh* transcription). (**c**) Chick leg antero‐posterior specification—cells that give rise to digits 1, 2 and 3 specified in same manner as in the chick wing (a), a parallel process of autocrine Shh signaling in cells of the polarizing region specifies positional values appropriate for digit 4 over 16 h (shown green to indicate derived from polarizing region cells). Note inverted commas indicate a digit forming with the character of a more‐anterior digit of the pattern

The signaling center, which became known as the polarizing region (or zone of polarizing activity—ZPA), and which specifies pattern across the antero‐posterior axis of the limb, was discovered in a series of experiments where mesenchyme tissue was grafted from the posterior margin of the early wing bud of one chick embryo to the anterior margin of a wing bud of a recipient embryo (Figure 3a, Saunders & Gasseling, 1968). This manipulation duplicated the normal pattern of three digits (1, 2 and 3) to result in mirror‐image patterns such as 3, 2, 1, 1, 2 and 3 (Figure 3a—note digits were designated 2, 3 and 4 at the time—see section 1.7). These results were consistent with the polarizing region producing a long‐range paracrine signal, or morphogen, which would provide cells with a positional value (Tickle, Summerbell, & Wolpert, 1975; Wolpert, 1969). Cells would interpret this positional information and use it to instruct their differentiation into the correct type of structure (i.e., the type of digit). Intensive investigation, mostly using the manipulation outlined in Figure 3a, revealed that the morphogen specifies antero‐posterior positional values in a concentration and time‐dependent manner. Thus, upon receiving increasing levels of signal for a longer duration, cells progress through positional values that are appropriate to specify a digit 1, then a digit 2 and finally a digit 3, with each of these “promotions” requiring 4 h (Honig, 1981; Smith, 1980; Smith, Tickle, & Wolpert, 1978; Tickle et al., 1975; Yang et al., 1997—reviewed in Tickle & Towers, 2017). It was also revealed that the morphogen regulated the production of an apical ectodermal ridge maintenance factor (Zwilling & Hansborough, 1956), which was later identified by work on the mouse limb to be encoded by the Bone Morphogenetic Protein (BMP) antagonist, *Gremlin1* (Zuniga, Haramis, McMahon, & Zeller, 1999). The apical ectodermal ridge is a thickening of the distal‐most epithelium of the limb that lies at the boundary between dorsal and ventral sides (Fernandez‐Teran & Ros, 2008), and which produces signals (later shown to be largely based on Fibroblast Growth Factors, FGFs), that are essential for outgrowth along the proximo‐distal axis (shown by blue lines in Figure 3, Cohn, Izpisua‐Belmonte, Abud, Heath, & Tickle, 1995; Fallon et al., 1994; Niswander, Tickle, Vogel, Booth, & Martin, 1993; Niswander, Jeffrey, Martin, & Tickle, 1994). Cells of the polarizing region were not predicted to express the apical ridge maintenance factor, and this provided an explanation for why grafts of the chick wing polarizing region made distally to a host wing bud, led to flattening of the immediately adjacent apical ectodermal ridge (Saunders & Gasseling, 1968, see also Saunders, 1977). Although the morphogen was demonstrated to specify antero‐posterior positional values, early evidence suggested that it was not required for the periodic formation of cartilage condensations. Thus, morphologically similar digits formed even if leg bud anterior mesenchyme (with the polarizing region removed) was disaggregated, then reaggregated into a pellet, and then placed in an ectodermal hull, which was then grafted to a host embryo (Pautou, 1973; Zwilling, 1964). If a polarizing region was grafted to such reaggregated limb buds, the digits that formed had more distinct morphologies (MacCabe & Saunders, 1971). These results were consistent with a self‐organizing Turing‐type mechanism—possibly based on reaction/diffusion—determining the number of digit condensations (Newman & Frisch, 1979; Wilby & Ede, 1975). The number of digit condensations depends on the width of the limb bud and the wavelength/periodicity of the self‐organizing mechanism. The polarizing region signal would provide the information required for each condensation to form with a particular morphology. Thus, the power of positional information and self‐organization co‐operating in embryonic patterning was realized from such early experimental work on chick limbs (Wolpert, 1989). There has been a recent resurgence on the study of self‐organization in limb development, particularly from work on the mouse limb (Raspopovic, Marcon, Russo, & Sharpe, 2014; Sheth et al., 2012, reviewed in Green & Sharpe, 2015).

The pivotal discovery that the morphogen encoded by the *Sonic hedgehog* (*Shh*) gene is secreted by the polarizing region, and when applied to the anterior margins of chick wing buds in the form of *Shh*‐expressing cells or recombinant Shh protein, could mimic the effects of polarizing region grafts (Riddle, Johnson, Laufer, & Tabin, 1993), paved the way for later work that examined the function of Shh in normal limb development. In limbs of the *Oligozeugodactyly* chicken, which develop in the absence of Shh signaling, digits fail to form in the wing, and all but the most‐anterior toe fails to form in the leg (Ros et al., 2003). These patterns of digit loss are comparable to those obtained following the genetic removal of *Shh* signaling in the fore‐limbs and hind‐limbs of mice (Chiang et al., 1996).

More recently, the ability to block Shh signaling, by administering cyclopamine (which blocks Shh signaling at the level of Smoothened) to the developing chick embryo, has given insights into the promotion of antero‐posterior positional values. Scherz et al showed that the earlier that cyclopamine was applied to chick embryos, the fewer posterior digits formed in wings—similar findings were also observed in legs (Scherz, McGlinn, Nissim, & Tabin, 2007). Earlier fate‐maps made by using lipophilic dyes that stain cell membranes (Vargesson et al., 1997), had shown that the digits of the chick wing bud are derived from cells located in the posterior half of the early bud. The subsequent fate mapping of the cells that give rise to the digits in the wings of chick embryos treated with cyclopamine, revealed the spatial and temporal process of positional value specification how this is integrated with growth (Figure 3b, Towers, Mahood, Yin, & Tickle, 2008; Towers, Signolet, Sherman, Sang, & Tickle, 2011). Thus, if Shh signaling is inhibited 4 h after the onset of *Shh* transcription, wings form with a single digit 1, at 8 h, wings form with digits 1 and 2, and finally at 12 h, wings form with digits 1, 2 and 3 (Figure 3b, Towers et al., 2008, 2011). In addition, it was revealed that Shh signaling promotes expansion of the digit‐forming field (Towers et al., 2008). Thus, the size of the digit‐forming field is determined at a stage corresponding to the bud shown on the far‐left in Figure 3b, and this is likely due to cells in this field responding to the initial burst of Shh signaling and rapidly up‐regulating the gene encoding the main receptor of Shh, *Ptch1* (Drossopoulou et al., 2000). Shh promotes further antero‐posterior growth of this “primed” digit‐forming area to provide enough tissue for the positional values appropriate for three digits to be specified—shown in the bud on the far‐right in Figure 3b (Towers et al., 2008). Shh signaling to this field then indirectly determines the length of the overlying apical ectodermal ridge (via induction of *Gremlin1* in adjacent mesenchyme) and this permits proximo‐distal outgrowth. The molecular basis by which a gradient of Shh signaling is translated into a transcriptional response in the developing limb has been determined (reviewed in Tickle & Towers, 2017). In brief, Shh signaling prevents the processing of the full‐length form of the Gli3 transcription factor into a repressor form and this event de‐represses genes required for antero‐posterior patterning. Thus, in the absence of Gli3, the limbs of mice form up to eight digits, showing that polydactyly is constrained by the active repression of the transcriptional response to Shh signaling (Litingtung, Dahn, Li, Fallon, & Chiang, 2002; te Welscher et al., 2002). Gli3 processing occurs in the primary cilia and the loss of this structure can therefore also cause polydactyly (reviewed in Bangs & Anderson, 2017). Indeed, the classical chicken mutants, *talpid^2^* and *talpid* ^3^, fail to produce primary cilia, and this results in loss of Gli3 function and polydactyly (Chang et al., 2014; Yin et al., 2009).

As in the chick wing, Shh is predicted to specify positional values in the mouse limb during early bud stages (Zhu et al., 2008), but how this is accomplished remains unclear (see section 1.9, reviewed in Tickle & Towers, 2017). Important work showed that the two most‐posterior digits of the mouse limb are entirely derived from the cells of the polarizing region, and therefore predicted to be specified by the length of time that cells are exposed to autocrine Shh signaling (Harfe et al., 2004). Long‐term fate maps have been subsequently made in which the polarizing regions of HH20 chick wing buds were replaced with polarizing regions excised from the wing buds of transgenic chick embryos that constitutively express Green Fluorescent Protein (GFP). The resulting sections showed that polarizing region cells contribute to the soft tissues running along the posterior margin of digit 3, but not to the digit skeleton (Towers et al., 2011) Figure 3b—polarizing region is green to represent GFP labeled cells). Short‐term fate maps of the HH20 chick wing polarizing region made by applying lipophilic dyes also showed a contribution to digit 3, although it was unclear which of the cell types were labeled (Tamura, Nomura, Seki, Yonei‐Tamura, & Yokoyama, 2011). The application of cyclopamine to chick embryos with GFP‐expressing polarizing regions confirmed that promotion by paracrine Shh signaling occurs in adjacent cells (Figure 3b; Towers et al., 2011). Further grafts, using the same technique, accurately mapped the positions at which cells give rise to the three digits of the chick wing, and these correspond to the positions shown in the limb bud on the far‐right of Figure 3b (Fisher et al., 2011).

### 1.4 Chick leg digit patterning

Less attention has been paid to understanding how the pattern of four chick leg digits is specified. Early experiments showed that grafts of a leg bud polarizing region made to the anterior margin of the leg bud of a host embryo duplicated the pattern of digits (Summerbell & Tickle, 1977). However, an intriguing finding was that grafts of the chick leg polarizing region made to the anterior margin of a host chick wing, as well as duplicating the wing digits, also often produced a leg digit (Summerbell & Tickle, 1977). This was explained by the demonstration that a GFP‐expressing leg polarizing region graft, made in place of the normal leg polarizing region, gives rise to the most‐posterior digit—digit 4 (Towers et al., 2011, Figure 3c). To understand how antero‐posterior positional values are specified in the chick leg, similar experiments to those discussed in the previous section were performed, in which cyclopamine was applied to embryos, and the fate of the grafted GFP‐expressing polarizing region determined (Towers et al., 2011). This revealed that digits 1, 2 and 3 are specified by paracrine Shh signaling in the same manner as digits 1, 2 and 3 in the chick wing (Towers et al., 2011, Figure 3b,c). In addition, it was shown that digit 4 is specified in parallel through the full range of digit positional values over 16 h (Figure 3c). Thus, the simultaneous process of paracrine and autocrine specification can explain the different digit patterns that are obtained when cyclopamine is administered to chick embryos at a series of stages (Towers et al., 2011, Figure 3c). Therefore, antero‐posterior positional values are specified in the leg bud by an early stage, but this takes 4 h longer than it does in the wing bud. An interesting facet of chick leg digit development is that the number of phalanges is directly related to the length of time that cells are exposed to Shh signaling and also their position in the bud. Thus, the number of phalanges in a digit increases by one for every promotion (4 h exposure to Shh signaling), and also by one going from anterior to posterior across the bud toward the source of Shh signaling (see also section 1.9).

### 1.5 Interpretation of positional values into digit morphology

The fact that, depending on the length of exposure to paracrine or autocrine Shh signaling, equivalently positioned cells can give rise to any digit of the chick wing and leg (Figures 3b,c), is consistent with the idea that antero‐posterior positional values specified by Shh signaling can determine all aspects of digit morphology, including phalange number and digit length. However, the relationship between antero‐posterior positional information and digit morphology in some amniotes is not as apparent as it is in birds, and this will be discussed in section 1.9. Indeed we know little about how antero‐posterior positional values are recorded and then interpreted later in development. Important work on chick limbs revealed that the positional information specified in the early limb bud could be altered at late digit condensation stages. Thus, the application of signals, such as BMPs (Dahn & Fallon, 2000), Shh (Sanz‐Ezquerro & Tickle, 2003) and FGFs (Casanova, Badia‐Careaga, Uribe, & Sanz‐Ezquerro, 2012) at digit condensation stages, can alter the number of phalanges in a digit. However, it should be noted the digit condensations of the chick leg are more labile to BMP signals than the condensations of the chick wing (Dahn & Fallon, 2000), and that there are differences between the different chick wing digits themselves in response to FGF signals (Casanova et al., 2012). This is likely to reflect that independent signaling pathways operate in different digit condensations, but how they are established downstream of Shh signaling remains unclear (see also section 1.9). However, one interesting finding is that the level of BMP signaling across the antero‐posterior axis of the chick leg at digit condensation stages mirrors the concentration of Shh predicted to specify antero‐posterior positional values at an earlier stage—progressively increasing in the condensations of digit 1 through to digit 3 (specified by paracrine Shh signaling), with the lowest levels in the condensation of digit 4 (specified by autocrine Shh signaling, Suzuki, Hasso, & Fallon, 2008). It should be noted that the length of a digit generally correlates with the duration that *Fgf8* is expressed in the apical ectodermal ridge and that the longest digits do not always have the most phalanges. For instance, in the chick leg, *Fgf8* persists for the longest duration in the apical ectodermal ridge overlaying the condensation that gives rise to the longest digit, digit 3, which has one fewer phalanx than digit 4 (Seki et al., 2015). Therefore, it is unclear how the periodicity of phalanx formation within a particular digit is controlled and this is likely to involve complex interactions between BMP signaling from the interdigital mesenchyme and FGF signaling from the apical ectodermal ridge operating downstream of Shh signaling (see Huang et al., 2016 for recent work on the mouse limb). Furthermore, additional later events during cartilage and bone differentiation might affect final digit length and morphology.

### 1.6 The chick leg is an excellent model for evolutionary studies

Although studies on the bird wing have greatly enriched our knowledge of limb development, the fact that it is a very derived structure somewhat restricts the extent to which it can inform us on wider evolutionary questions. However, the bird leg presents such an opportunity, because as mentioned already, it is of special interest because its four digits have retained the basal amniote phalangeal pattern (Figure 1). The finding that digit 4 of the chick leg, mouse fore‐limb and mouse hind‐limb are fully derived from the polarizing region, is likely to indicate that this is an ancestral condition. This is supported by the fact that none of the three digits of the chick wing are derived from the polarizing region (Figure 3a). We can also speculate that the pattern of 5′ *Hoxd* expression, in which *Hoxd9–13* are expressed in the condensations of cells that give rise to digits 2, 3, 4 and 5, while *Hoxd13* is the only 5′ *Hoxd* gene expressed in the condensation that gives rise to digit 1, is also an ancestral character, since comparable patterns have been reported in the limb buds of chicks and mice (Galis, Kundrat, & Metz, 2005; Vargas & Fallon, 2005).

**Figure 4:**
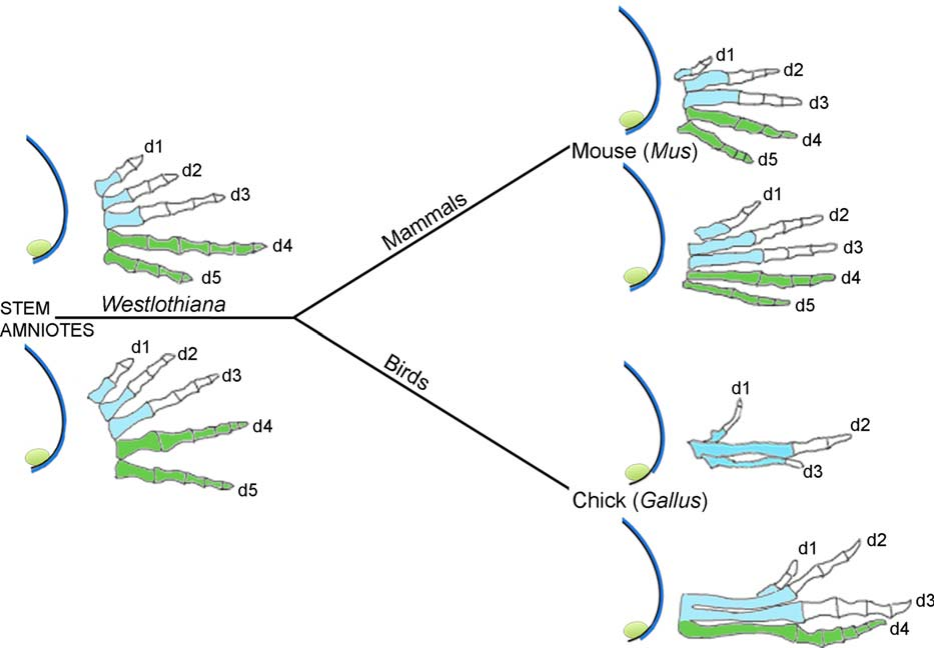
Evolution of the polarizing region cell lineage. Polarizing region gives rise to digits 4 and 5 of the mouse fore‐limb/hind‐limb (shown by green digits), no digits of the chick wing, and digit 4 of the chick leg. Prediction that digits 4 and 5 were derived from polarizing region of the limbs of a stem amniote such as *Westlothiana*

Based on the above considerations, it is likely that the positional values of digits 1, 2, 3 and 4 of the limbs of a stem amniote such as *Westlothiana* were specified in a similar fashion to the digits of the chick leg (Figure 4). In addition, it is likely that digits 4 and 5 were derived from the polarizing region, as is the case in the mouse limb (Figure 4). It is unclear, however, if a model involving the promotion of positional values is involved in patterning digits 1, 2, 3 and 4 of the mouse limb, and therefore, if this potential ancestral mechanism has been conserved in mammals (discussed in section 1.9). This has largely arisen from the difficulty in interpreting the patterns of digits that result from the truncation of Shh signaling in the mouse limb, and also from assuming that phalangeal number provides a direct read‐out of antero‐posterior positional values, as it does in the chick leg (see section 1.9).

### 1.7 Evolution of theropod/bird digit patterns

It is widely accepted from the fossil record that digit 5 was lost in the hind‐limbs of theropod dinosaurs that gave rise to birds (Figure 2). The mechanism that resulted in loss of this digit is unclear and will be speculated upon in section 1.8. However, despite the fossil record appearing to show that the theropod hand/bird wing was reduced to three digits by a simple process of posterior digit loss (Figure 2), this has in fact been a contentious issue in evolutionary/developmental biology. The crux of the matter is the alternative suggestion that the digit condensations of the avian wing arise from conserved positions along the antero‐posterior axis, and that these positions are 2, 3, and 4, rather than 1, 2, and 3 (Burke & Feduccia, 1997). The principle argument to support the identification of the avian wing digits as 2, 3 and 4 is that rudimentary condensations have been reported in positions lying both posterior and anterior to the true digit condensations in the embryonic limbs of several species of bird (Burke & Feduccia, 1997; Feduccia & Nowicki, 2002; Hinchliffe, 1977; Larsson & Wagner, 2002; Welten, Verbeek, Meijer, & Richardson, 2005). In addition, it has been postulated that a conserved “primary axis of condensation” is found in the limbs of all amniote species, and which runs through the ulna and into digit 4 (Burke & Feduccia, 1997). This is proposed to impose a developmental constraint on the limbs of all tetrapod species—even ones that have undergone substantial digit loss—to retain a digit 4. Therefore, it has been suggested that digits 1 and 5 were lost during theropod hand evolution, and not digits 4 and 5 (Xu et al., 2009). However, as already discussed, there is sparse evidence for this in the fossil record, other than in the limbs of the derived ceratosaur, *Limusaurus*, which was not a transitory species in the evolution of birds, as discussed previously in section 1.2 (Figure 2, Xu et al., 2009). In addition, RNA sequencing of developing chick wing and chick leg digit condensations, revealed a clear transcriptional signature that unites digit 1 of both limbs (Wang, Young, Xue, & Wagner, 2011) —thus, adding to previous findings, that the cells which give rise to digit 1 have a unique *5*′*Hoxd* code, expressing only *Hoxd13*. Therefore, the weight of both paleontological and molecular evidence suggests that digit 1 is present in the wings of birds, and therefore that the digits are 1, 2, and 3. However, it should be noted that some researchers still adhere to the identification of the bird wing digits as 2, 3, and 4 (de Bakker et al., 2013).

Several solutions have been proposed for resolving the apparent discrepancy between paleontological/molecular and embryological data and have been discussed in depth elsewhere (Xu & Mackem, 2013). Here, it will be discussed how two main hypotheses stand up in respect to what we know about how antero‐posterior positional values are specified in chick limbs as outlined in section 1.3. The “frame shift” hypothesis (Wagner & Gauthier, 1999) states that digits identified as digits 1, 2 and 3 have “shifted” position, and now arise from condensations found in positions 2, 3 and 4 of the avian wing bud, thus conserving the primary axis (reviewed in Young, Bever, Wang, & Wagner, 2011) —see other related models that all involve conservation of the axis (Xu & Mackem, 2013). It is unclear when in the transition between theropod dinosaurs and modern birds a frame‐shift is predicted to have occurred, but presumably, it must have occurred before the disappearance of the rudimentary digit 4, which was last seen in the fore‐limbs of theropods such as *Dilophosaurus* (Figure 2). It is difficult to conceive how it could have occurred later, as theropods such as *Allosauru*s had already lost digit 4. An alternative solution to explain the loss of digits in the theropod fore‐limb is the “axis shift” hypothesis (Chatterjee, 1998; Garner & Thomas, 1998; Shubin, 1994). In this model, digits 1, 2, and 3 arise from condensations located in positions 1, 2, and 3 in the avian wing bud, and digits 4 and 5 have been lost, as shown in the fossils of early theropods (Figure 2). Two main conditions are required for an axis shift to have occurred: first that the condensation that lies anterior to the digit 1 condensation is not a condensation of a digit, but of another vestigial structure called a prepollex (Welten et al., 2005); second, that there is not a constraint on a primary axis of condensation in the digit 4 position, and upon the loss of this digit, the axis was simply “shifted” anteriorly into the digit 3 position (Chatterjee, 1998; Garner & Thomas, 1998; Shubin, 1994).

**Figure 5:**
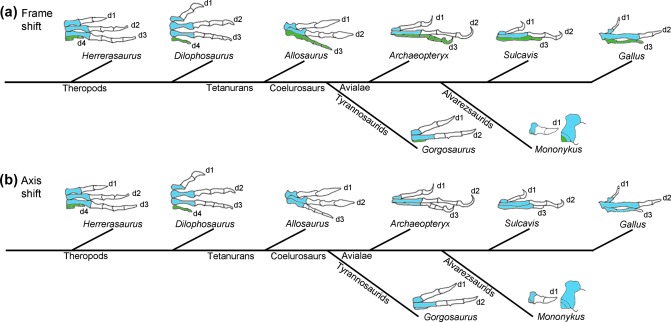
Models of theropod fore‐limb digit evolution. **(a**) Frame‐shift model—In the transition from a theropod limb with four digits (i.e., *Dilophosaurus*) to a limb with three digits (i.e., *Allosaurus*), the primary axis of condensation in cells of the polarizing region “shifted frame” and went from producing a rudimentary digit 4 to producing a robust digit 3 (shown by green digits). In cases of further skeletal element loss/reduction in tyrannosaurids and alvarezsaurids, the primary axis of condensation in the polarizing region gave rise to a rudimentary metacarpal in both *Gorgosaurus* limbs (colored green) and *Mononykus* limbs (green in enlarged area). (**b**) Axis‐shift model—during theropod evolution the primary axis of condensation in the polarizing region failed to produce a digit 4 (shown colored green in *Allosaurus* and *Dilophosaurus* limbs), and upon loss of this structure, the primary axis of condensation “shifted” and produced a digit 3. Further loss of posterior structures in tyrannosaurids and alvarezsaurids resulted in further shifts of the primary axis of digit condensation to produce a digit 2 in *Gorgosaurus* limbs and a digit 1 in *Mononykus* limbs

Figure 5 shows how the frame shift and axis shift hypotheses could explain the transition in limb anatomies from early theropod dinosaurs to modern birds. If we start with the prediction that digit 4 was derived from the cells of the polarizing region of the ancestral amniote limb bud (Figure 4), and hence the limb buds of the earliest theropods (Figure 5), if a frame‐shift occurred at some point during coelurosaur evolution, the digit 4 condensation—upon the point of regression—would then have given rise to a digit 3 (Figure 5a). Indeed, much weight has been given to the fact that the inhibition of Shh signaling in chick wing buds can cause a “frame‐shift” that results in two digits, 1 and 2, arising from positions 2 and 3 (Salinas‐Saavedra et al., 2014; Vargas & Wagner, 2009), in other words truncating the promotion of antero‐posterior positional values as shown in an earlier study (Towers et al., 2008, Figure 3b). This is accompanied by a posterior shift in the expression of 5′ *Hoxd* expression (Salinas‐Saavedra et al., 2014; Vargas & Wagner, 2009). However, the relevance of this extrapolation is unclear as it involves a frame‐shift occurring in a bird wing with three digits, which is the evolutionary endpoint considered here. On the other hand, were a frame‐shift to occur in a limb with four digits—as predicted for theropod fore‐limbs—this then implies that the most‐posterior digit of a limb with three digits will be derived from the polarizing region (Towers et al., 2011, Figures 5a and 3c). As discussed in section 1.3, the polarizing region of the chick wing does not contribute to the digit skeleton; therefore, it appears unlikely that there has been a posterior shift in the positions from which the digits arise in the limb bud during theropod hand/bird wing evolution. However, it could be argued that the position of the polarizing region has itself “shifted” in the bird wing, and should not be used to position the primary axis (Xu & Mackem, 2013). This seems unlikely, since the position of the polarizing region in the chick wing bud and the chick leg bud is indistinguishable at the time at which antero‐posterior positional values are specified (Figure 3b,c). In addition, since three digits are specified in cells adjacent to the polarizing region of the chick wing, chick leg, mouse fore‐limb and mouse hind‐limb, this indicates that digit positions have been conserved throughout evolution in respect to the polarizing region (Harfe et al., 2004; Towers et al., 2011). Indeed, the polarizing region itself could be considered to position the primary axis of growth in the limb, as it constitutes an antero‐posterior boundary, which, by intersecting with the dorso‐ventral boundary, acts to maintain the apical ectodermal ridge (Meinhardt, 1983). The positioning of a primary axis of growth in relation to Hedgehog producing cells is also found in the *Drosophila* wing disc (Meinhardt, 1983; Varjosalo & Taipale, 2008), and in the blastemas of regenerating amphibian limbs (Nacu, Gromberg, Oliveira, Drechsel, & Tanaka, 2016), thus suggesting that this a general aspect of appendage development. In summary, both developmental and paleontological data support the idea that the primary axis of the bird wing is in the digit 3 position (Figure 5b). It is noteworthy that an axis shift into the digit 2 position is considered to have occurred in some amphibian limb buds, thus indicating that this mechanism is not developmentally constrained (Shubin & Alberch, 1986).

In terms of theropod limb evolution, it is of interest if one considers the frame‐shift and axis‐shift hypotheses in cases of further digit loss that occurred in the fore‐limbs of some tyrannosaurids and alvarezsaurids (Figure 2). Thus, in the frame‐shift model, the primary axis is expected to have terminated prematurely as a metacarpal in *Gorgosaurus* fore‐limbs, and a vestigial metacarpal in *Mononykus* fore‐limbs (Green in enlarged area ‐Figure 5a). If then the primary axis can terminate as a rudimentary structure, this could imply that it could also terminate as the vestigial structure that forms in the fourth digit position of the bird wing (Hinchliffe, 1977). This would further support the idea that a frame‐shift has not occurred during theropod/bird evolution, but it would also suggest that the primary axis has not shifted position. Thus, the apparent shift of the primary axis into the digit 3 position in bird wings, the digit 2 position in *Gorgosaurus* fore‐limbs, and the digit 1 position, in *Mononykus* fore‐limbs would be cryptic (Figure 5b), and would only be a consequence of the failure of more‐posterior structures to completely develop along the primary axis.

### 1.8 Basis of posterior digit loss in theropod limbs

In this section, potential mechanisms that could account for the loss of posterior digits in theropod/bird limbs will be discussed. One way to begin to address this is to look for differences in the development of the posterior part of the limb buds of species that produce different numbers of posterior digits. A clear difference is the extent to which the apical ectodermal ridge extends posteriorly, in relation to the number of digits that the polarizing region produces—none in the chick wing, one in the chick leg and two in mouse limbs (Pickering & Towers, 2016). As mentioned earlier, the apical ectodermal ridge is required for the development of the underlying mesenchyme, thus implicating it in the ability of the polarizing region to form digits. Indeed, early experiments on the chick wing revealed that one of the first effects of excising the apical ectodermal ridge was apoptosis in a band of underlying mesenchyme (Cairns, 1975). Two regions—originally called necrotic zones—are found at the anterior and posterior margins of the chick wing bud, lying proximal to each end of the apical ectodermal ridge (Saunders & Gasseling, 1962). However, these regions of apoptosis are reduced in the chick leg bud, and absent in mouse limb buds (Fernandez‐Teran, Hinchliffe, & Ros, 2006). Therefore, posterior digit loss is related to the length of the apical ectodermal ridge and also to the extent of apoptosis.

The first chick study to support the idea that the absence of the apical ectodermal ridge—or the signals it produces—could result in loss of posterior digits, involved implanting FGF‐soaked beads into the posterior part of the wing bud (Nikbakht & McLachlan, 1999). This experiment showed that a rudimentary digit could be generated posteriorly adjacent to digit 3 (Nikbakht & McLachlan, 1999). What factors therefore determine the posterior limit of the apical ectodermal ridge? As mentioned previously, when a chick wing polarizing region is grafted to the distal tip of another wing bud, it causes the overlying apical ectodermal ridge to flatten and regress (Saunders & Gasseling, 1968). Therefore, it is significant that equivalent grafts of a HH20 chick wing polarizing region, which do not normally give rise to a digit when grafted in place of a wing polarizing region, do so, when made in place of a leg polarizing region (Summerbell & Tickle, 1977; Towers et al., 2011). This suggests it is the refractoriness of the apical ectodermal ridge to a polarizing region signal that dictates the extent to which it persists posteriorly. Genetic studies in the mouse limb support this proposal and implicate Shh as the signal (Bouldin, Gritli‐Linde, Ahn, & Harfe, 2010). In addition, a recent study showed that the application of cyclopamine to the chick embryo, at HH20/21, could result in wings forming with four digits, often in patterns of 1, 2, 2 and 2 (Pickering & Towers, 2016, Figure 6a—the genesis of pattern will be discussed in the next section). The fourth digit of this pattern is derived from the cells of the polarizing region, and this is dependent on the presence of a posteriorly extended apical ectodermal ridge (Pickering & Towers, 2016). This finding shows that it is possible for a digit to develop from a condensation that normally regresses, thus potentially “shifting” the primary axis back into the digit 4 position—a condition last seen in in the fore‐limbs of basal theropods (Figure 5b). Further analyses showed that posterior apoptosis is undetectable and also that polarizing region cell proliferation is increased in cyclopamine‐treated wing buds that produce an additional posterior digit (Pickering & Towers, 2016). Therefore, the loss of posterior digits in theropod/bird limbs is likely to be a consequence of Shh inhibiting the formation of the overlying apical ectodermal ridge. However, it should be noted that Shh also intrinsically regulates cell proliferation and apoptosis independent of the apical ectodermal ridge, hinting at the complex regulation of these processes in posterior mesenchyme (Bastida, Sheth, & Ros, 2009; Chinnaiya, Tickle, & Towers, 2014; Sanz‐Ezquerro & Tickle, 2000).

**Figure 6:**
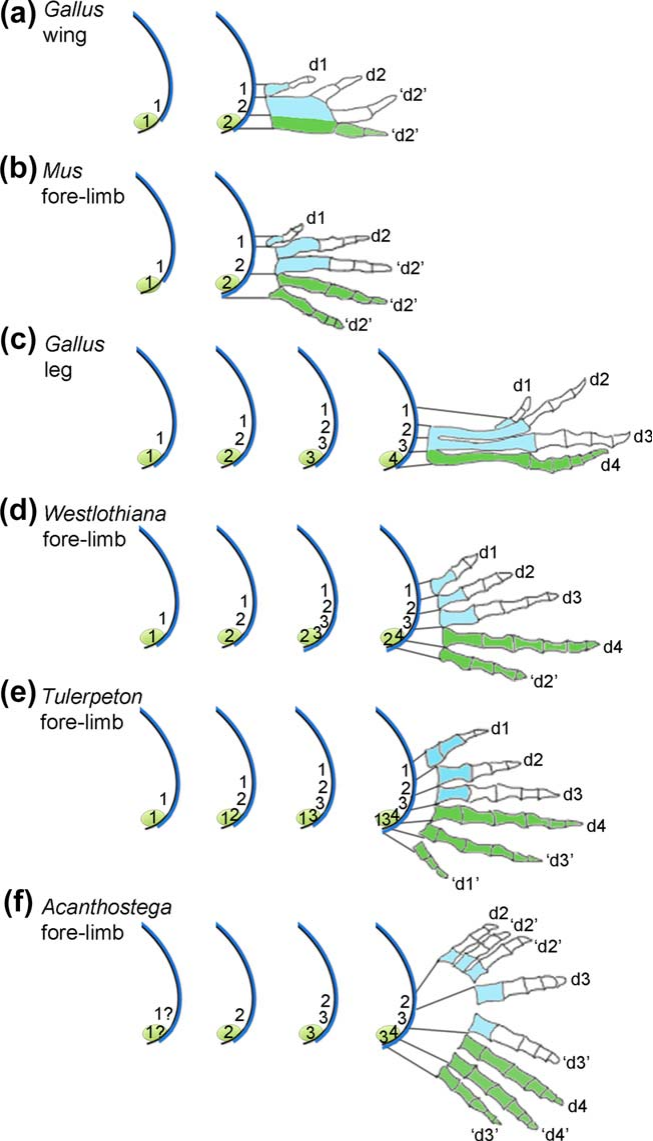
Models of tetrapod digit evolution. **(a**) Effects of inhibition of Shh signaling at HH20/21 on chick (*Gallus*) wing development (10 h of *Shh* transcription—see Figure 3b). Apical ectodermal ridge extends posteriorly and polarizing region (green) produces a “digit 2” (colored green). Note inverted commas indicate a digit that has the character (i.e., phalangeal number), but not necessarily the identity, of a more‐anterior digit of the pattern. Antero‐posterior expansion mediated by the apical ectodermal ridge results in a population of cells specified with equivalent antero‐posterior positional values producing two “digit 2s” by self organization (black lines). **(b**) Extrapolation of model shown in (a) onto mouse (*mus*) limb digit patterning. Cells adjacent to polarizing region become refractory to Shh signaling at an early stage and produce two “digit 2s” by self‐organization. Further extension of apical ectodermal ridge allows polarizing region cells, which are also refractory to Shh signaling, to produce two “digit 2s” by self‐organization. **(c**) Chick (*Gallus*) leg digit patterning—see legends of Figure 3b,c. **(d**) *Westlothiana* fore‐limb digit patterning. Digits 1, 2, 3 and 4 patterned the same as the digits of the chick leg (c). Posterior‐most polarizing region cells became refractory to Shh signaling at a very early stage and produced a “digit 2.” **(e**) *Tulerpeton* fore‐limb digit patterning. Digits 1, 2, 3 and 4 patterned the same as the digits of *Westlothiana* fore‐limbs (d). Polarizing region cells expanded sufficiently to give rise to three digits—most‐posterior cells became refractory to Shh signaling at a very early stage and produced a “digit 1”, adjacent cells became refractory later and produced a “digit 3.” (**f**) *Acanthostega* fore‐limb digit patterning. Positional values specified as in *Tulerpeton* fore‐limbs except “digit 1s” do not form (queried by question marks if this positional value was specified). Note numbers shown for digits are based on relationship to digits (in terms of phalangeal pattern) in other tetrapod limb patterns, not their numerical order in the pattern. Extensive antero‐posterior expansion allowed cells specified with equivalent positional values to give rise to either two or three digits by self organization (black lines)

### 1.9 Antero‐posterior positional values and the evolution of digit pattern

Several models have been proposed to explain how the digits of the mouse limb are specified, none of which provide a satisfactory mechanism for how this pattern evolved from the ancestral amniote limb (Tickle & Towers, 2017). However, a recent study in the chick wing has provided one mechanism and implies that the number of phalanges in a digit provides a direct read‐out of the extent to which cells responded to paracrine and autocrine Shh signaling (Pickering & Towers, 2016). As mentioned previously, the inhibition of Shh signaling in the chick wing bud by the application of cyclopamine to stage HH20/21 embryos can result in the formation of four digits, one of which arises from the cells of the polarizing region (Figure 6a, Pickering & Towers, 2016). At HH20/21, it is predicted that positional values appropriate for digits 1 and 2 have been specified (Figure 3b). Analyses of the developing wing buds showed that the loss of Shh signaling, specifically at this stage, causes the apical ectodermal ridge to extend over the polarizing region (Figure 6a, Pickering & Towers, 2016). This has two effects: the apical ectodermal ridge maintains polarizing region proliferation and suppresses posterior apoptosis allowing it to form a “digit 2” as discussed previously. In addition, it facilitates expansion of the wing bud along the antero‐posterior axis, which allows cells adjacent to the polarizing region to produce two “digit 2s” by self organization (Figure 6a, Pickering & Towers, 2016—compare with promotion of positional values in normal chick wings—Figure 3b). As mentioned in section 1.3, the widening of the bud in the presence of Shh signaling normally provides enough tissue for self‐organization to produce two digits, digits 2 and 3. Although speculative, the extrapolation of a similar mechanism depicted in Figure 6a onto mouse limb development could provide an explanation for how digits 2, 3, 4 and 5 each form with three phalanges (Figure 6b, Pickering & Towers, 2016). For this to occur, it is predicted that apical ectodermal ridge permits two digits to form from the polarizing region. Two aspects of antero‐posterior patterning in mouse limbs are consistent with the model outlined in Figure 6b: first, the prediction that antero‐posterior positional values are specified at a very early stage of development (Zhu et al., 2008‐ perhaps even earlier than in normal chick wing development); and second, the fact that cells do not respond to Shh signaling in a graded manner across the antero‐posterior axis as predicted in a classical positional information model (Ahn & Joyner, 2004).

As mentioned previously, if we consider a potential model for patterning digits 1, 2, 3 and 4 of the ancestral amniote limb, based on the chick leg, there is a clear correlation—running from anterior to posterior—between the number of phalanges in a digit, and the degree to which cells responded to either paracrine and autocrine Shh signaling—increasing by one phalange for each promotion (Figure 3c). However, this relationship does not appear to exist in the limbs of most species, including chick wings in which digits 2 and 3 are truncated and lack a terminal phalanx (Casanova et al., 2012), and also in mammalian limbs (Figure 1). This makes it difficult to understand how digit patterns are specified, and thus how they evolved. Therefore, although antero‐posterior positional values could be specified by the same parameters in all tetrapod limbs, it is the differences in their interpretation that results in digits forming with different numbers of phalanges (see section 1.5). For instance, digits 1 and 2 of the duck wing form a terminal phalanx, but only digit 1 of the chick wing forms one (see Casanova & Sanz‐Ezquerro, 2007 for further discussions on digit tip formation). In addition, if one considers the fins of some cetaceans such as dolphins, in which digits can have up to fourteen phalanges (Richardson & Oelschlager, 2002), the interpretation of antero‐posterior positional values might involve prolonging FGF signaling by the apical ectodermal ridge. Alternatively, such late morphogenetic events could occur independently of earlier positional information specified by Shh.

Nonetheless, a model in which the number of phalanges in a digit directly provides a read‐out of the degree to which cells responded to paracrine and autocrine Shh signaling, gives us an opportunity to predict how some diverse patterns that have appeared in the fossil record were specified. For instance, if we consider the fore‐limbs of *Westlothiana*, and assume that a positional information model—as described for the chick leg (Figure 6c) —was sufficient to pattern digits 1, 2, 3 and 4, then we are left explaining how the positional value of a fifth digit with three phalanges was specified (Figure 6d). One possibility is that the most‐posterior cells became refractory to Shh signaling at a point appropriate to specify the positional value of a “digit 2.” In support of this proposal, polarizing region cells, which give rise to digit 5 of the mouse limb, become refractory to Shh signaling at a very early stage of development (Ahn & Joyner, 2004). Interestingly, the refractory nature of Shh producing cells to Shh signaling appears to be a general feature in development, and occurs in such diverse systems as the ventral part of the neural tube (Ribes et al., 2010) and the posterior part of the *Drosophila* wing disc (Varjosalo & Taipale, 2008). A similar model could be applied to the fore‐limbs of *Tulerpeton*. However, this requires that the sixth digit in this pattern arose because the cells of the polarizing region expanded further to allow an extra condensation to form by self‐organization, and that these cells, being very posterior, became refractory to Shh signaling at an even earlier stage than their neighbors, at the point at which they were specified with positional values appropriate to specify a “digit 1” with two phalanges (Figure 6e). Interestingly, a mechanism such as this suggests that Shh signaling specifies digits in basal tetrapod limbs with fewer phalanges, towards the anterior, by a traditional gradient of paracrine Shh signaling, and towards the posterior, by a gradient of refractoriness to autocrine Shh signaling. Therefore, the refractoriness of posterior mesenchyme cells and the apical ectodermal ridge could be linked, and this would allow the formation of additional posterior digits with progressively more‐“anterior” character. Even if one considers the digits of the fore‐limbs of *Acanthostega*, a similar pattern to the ancestral amniote digit pattern can be made out, with digits forming with more phalanges towards the posterior, but again, the very posterior digit having fewer phalanges (Figure 6f). If one speculates that adjacent digits with the same number of phalanges in this pattern were derived by self‐organization from cells specified with equivalent positional values (Figure 6f), this then superficially combines a positional information model, such as the one for chick leg (Figure 6c), with a model in which self‐organization dominates, as proposed for the mouse limb (Figure 6b). In order for the limb buds of *Acanthostega* to have produced additional anterior digits, one possibility is that Gli3 was not functional following the specification of antero‐posterior positional values, and that this facilitated excessive limb bud widening, similar to limb buds of mice without Gli3 activity (Litingtung et al., 2002; te Welscher et al., 2002). Therefore, repression by Gli3 during the evolution of later amniote limbs could have contributed to constraining polydactyly and thus maintaining the pentadactyl pattern.

## 2 CONCLUSIONS

The developing wings and legs of chicken embryos have provided valuable insights into the mechanisms of digit patterning. This has established a solid foundation for discussing how these digit patterns and some other tetrapod digit patterns could have arisen during evolution. The difficulty lies in that mechanisms of limb evolution are only based on conjecture and that many parameters are likely to remain unknown.

The challenge is to establish new model species with diverse digit patterns in their limbs, in order to gain further insights into the mechanisms of antero‐posterior patterning, which can then enhance the predictions made regarding the evolution of digit patterns. Recent progress has been made in this area following studies on mammalian limbs, both in showing that digit loss could have been caused by changes in the response of cells to the Shh signaling gradient in cows, and by increased cell death in camels, horses and jerboas (Cooper et al., 2014; Lopez‐Rios et al., 2014). In addition, a major gap in our understanding resides in our lack of knowledge of the molecular mechanisms that result in the formation of particular type of digit in a particular position. Ever emerging genomics techniques, that can detect small quantitative transcriptional changes and that determine the promoter occupancy of key developmental genes, such as *5*′*Hoxa/d* transcription factors, could help uncover differences in spatial and temporal gene expression, which relate to changes in digit anatomy between species.
